# Supplementary material for: RNase H2, mutated in Aicardi‐Goutières syndrome, promotes LINE‐1 retrotransposition
Source: EMBO J. 2018 Jun 29;37(15):e98506. doi: 10.15252/embj.201798506 (PMC6068448; doi:10.15252/embj.201798506)
Supplement: Supplementary file 1 — Appendix [file EMBJ-37-e98506-s001.pdf]

APPENDIX

RNase H2, mutated in Aicardi-Goutières syndrome, promotes LINE-1 retrotransposition

Maria Benitez-Guijarro, Cesar Lopez-Ruiz, Žygimantė Tarnauskaitė, Olga Murina, Mahwish Mian Mohammad, Thomas C. Williams, Adeline Fluteau, Laura Sanchez, Raquel Vilar-Astasio, Marta Garcia-Canadas, David Cano, Marie-Jeanne H. C. Kempen, Antonio Sanchez-Pozo, Sara R. Heras, Andrew P. Jackson, Martin A. M. Reijns* and Jose L. Garcia-Perez*

Contents:

Appendix Figure S1. Reduced LINE-1 retrotransposition in RNase H2 null HCT116 p53$^{-/-}$ cells

Appendix Figure S2. RNase H2 activity is dispensable for LTR-retroelement and DNA-Transposon activity

Appendix Figure S3. No increased mutation rate in *de novo* LINE-1 insertions in RNase H2 null cells

Appendix Figure S4. Increased RNase H activity against RNA:DNA heteroduplexes in RNASEH2A-KO HeLa cells complemented with human RNase H1

Appendix Figure S1

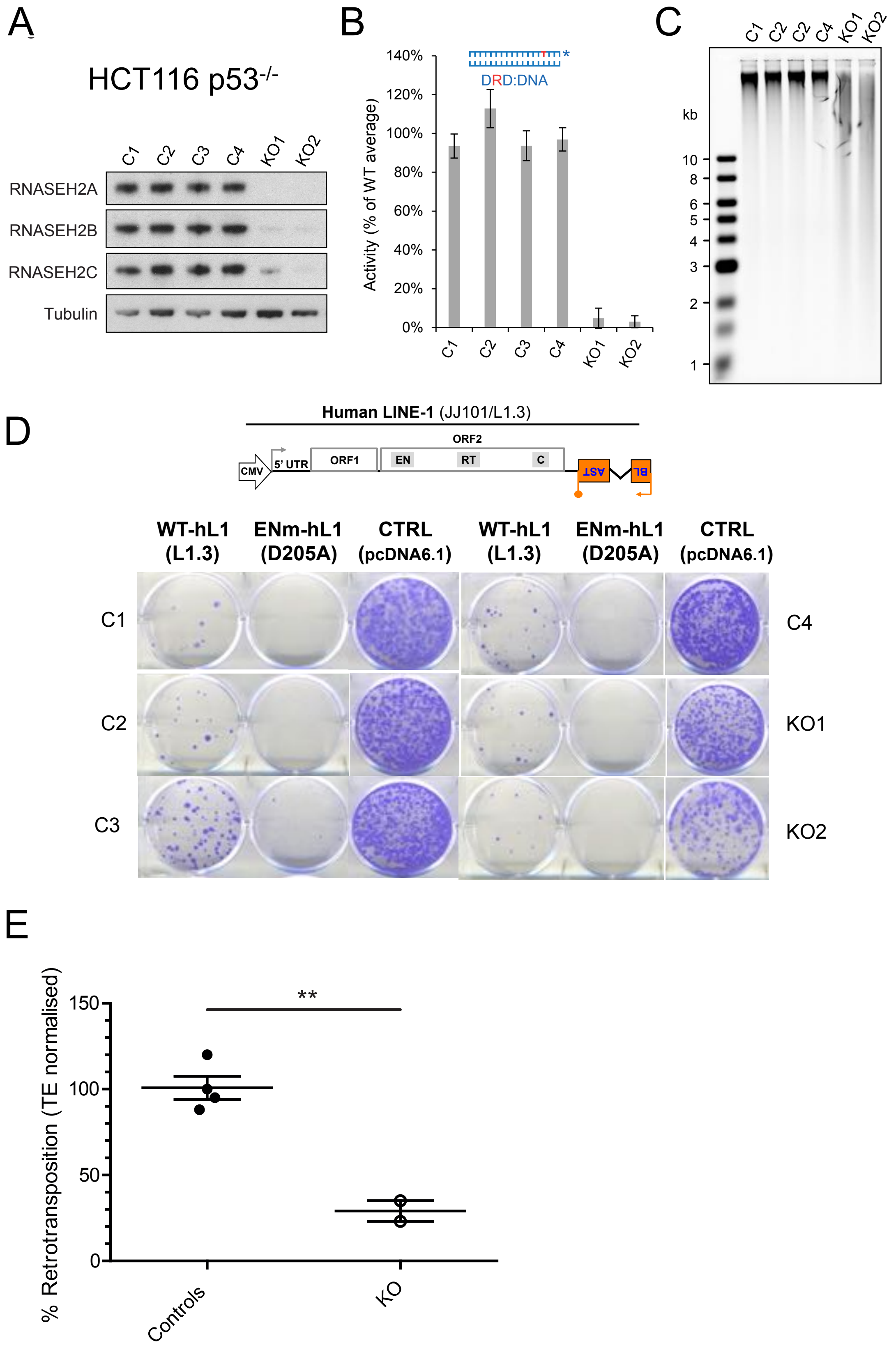

Appendix Figure S1. Reduced LINE-1 retrotransposition in RNase H2 null HCT116 p53$^{-/-}$ cells

A. Western blot analysis shows absence of RNASEH2A and reduced RNASEH2B and C in RNASEH2A-KO clones (KO1, KO2), compared to control clones (C1-4). Tubulin was used as a loading control. See also Source data.

B. RNase H assay shows absence of activity against single embedded ribonucleotides in KO clones, compared to control cells. Mean ± SD for n= 3 independent experiments.

C. High levels of genome embedded ribonucleotides in HCT116 p53$^{-/-}$ RNASEH2-KO clones. Genomic DNA was isolated from control and KO clones, RNase H2 treated and separated by alkaline gel electrophoresis. Smaller fragments indicate more genome embedded ribonucleotides.

D. Schematic of plasmid JJ101/L1.3, and representative retrotransposition and toxicity assays conducted in HCT116 p53$^{-/-}$ control clones (C1-4), and in two RNASEH2A-KO clones (KO1 and KO2). Cells were transfected with active human LINE-1 (WT-hL1, element L1.3), RT-mutant LINE-1 (RTm-hL1, D702A), or a toxicity control vector (CTRL, pcDNA6.1).

E. Quantification of L1-WT retrotransposition, with average retrotransposition in control cells set to 100% for comparison, shows reduced activity in RNase H2 null cells. Dots represent the mean of 3 technical replicates for individual clones. Lines indicate the mean of n=4 biological replicates for controls (C1-4) and n=2 for KO clones (KO1, 2) ± SEM (representative of 5 independent experiments). t-test, **, $p<0.01$

Appendix Figure S2

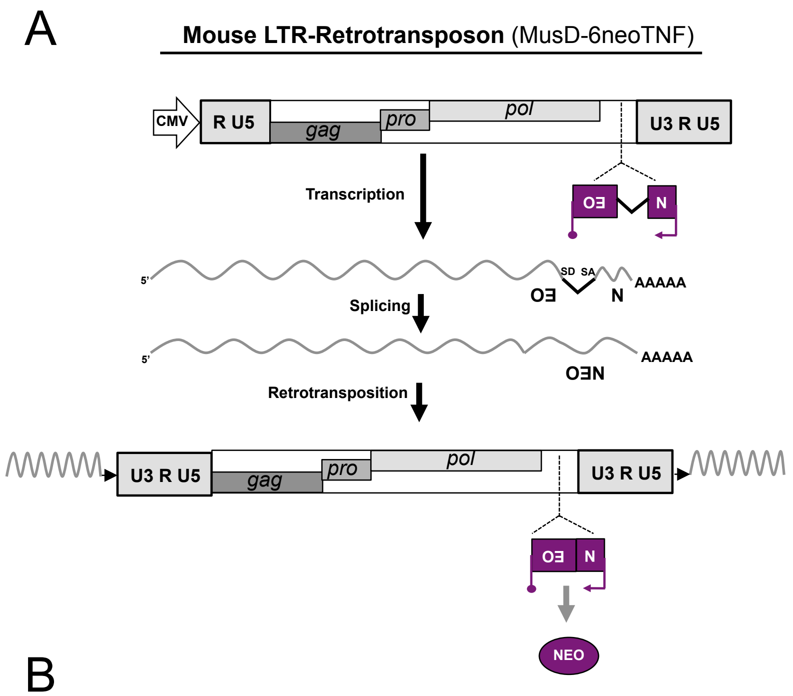

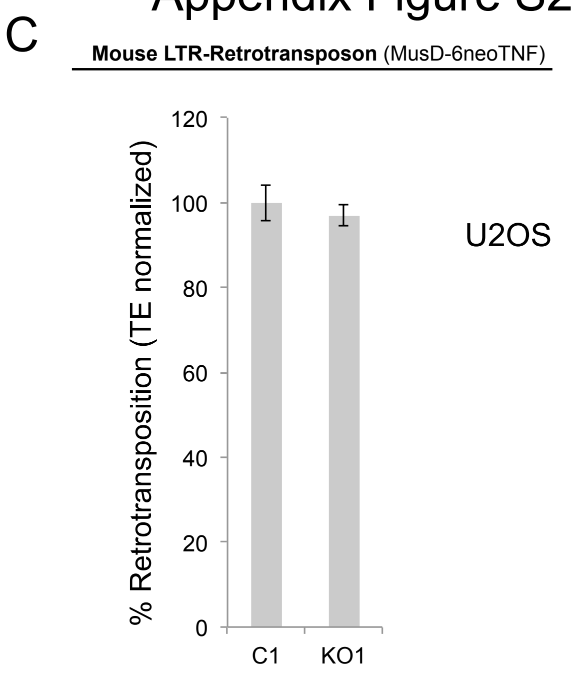

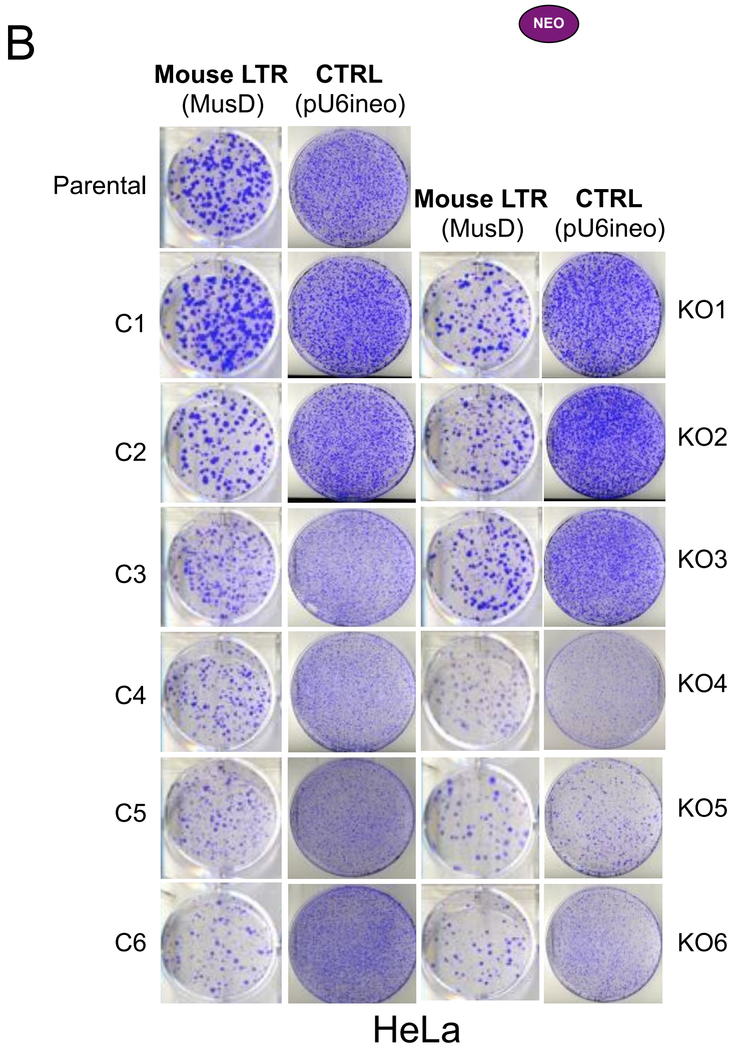

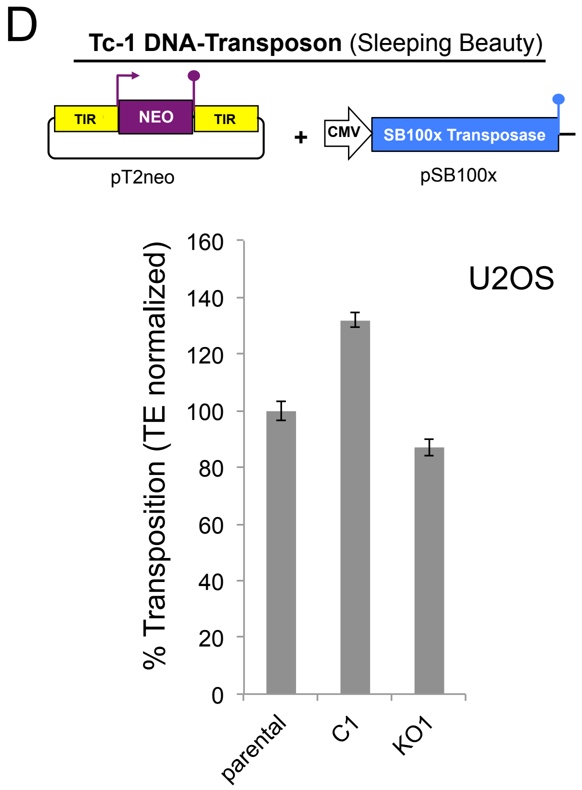

Appendix Figure S2. RNase H2 activity is dispensable for LTR-retroelement and DNA-Transposon activity

A. Schematic of a neo^{TNF} tagged MusD mouse LTR-retrotransposon. The relative position of the *gag*, *pro* and *pol* genes is indicated. The purple box with a backward NEO label depicts the retrotransposition indicator cassette neo^{TNF}, and the purple arrow and lollipop indicate the presence of a promoter and polyadenylation signal respectively. Upon transcription from the CMV promoter located upstream of the MusD element, this mRNA can be spliced by canonical *cis*-splicing and undergo a round of LTR-retrotransposition, resulting in the activation of the neo^{TNF} reporter and subsequent translation of the neomycin phosphotransferase protein (purple oval with white NEO label). In the LTR-retrotransposition event shown in the bottom, the black arrows indicate the presence of TSDs flanking the MusD insertion.

B. Representative results of LTR-retrotransposition assays in HeLa control (C1-6) and RNASEH2A KO (KO1-6) clones. Labels indicate if cells were transfected with a tagged active mouse MusD element (MusD) or with the toxicity control plasmid (pU6ineo).

C. Quantification of LTR-retrotransposition assays in U2OS control (C1) and RNASEH2A-KO1 clones. Mean ± SD for n=3 technical replicates (representative of 3 independent experiments).

D. Schematic of the two plasmids used in the Sleeping Beauty transposition assay. The purple box with a NEO label depicts the neo expression cassette. Underneath, quantification of SB assays in U2OS cells. Mean ± SD for n=2 technical replicates (representative of 3 independent experiments).

Appendix Figure S3

A

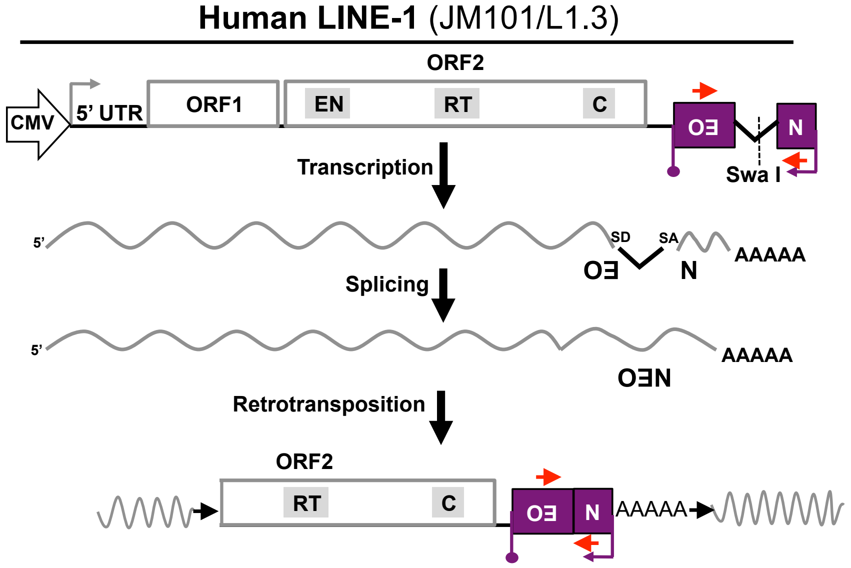

B

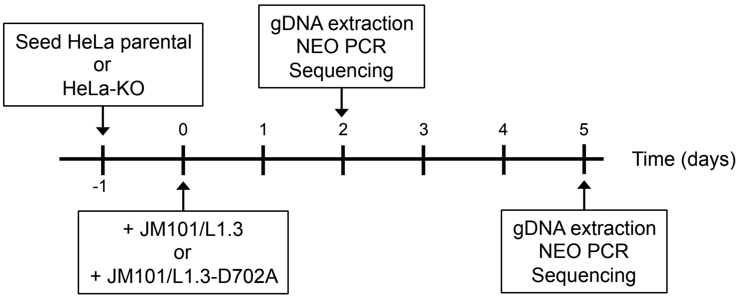

C

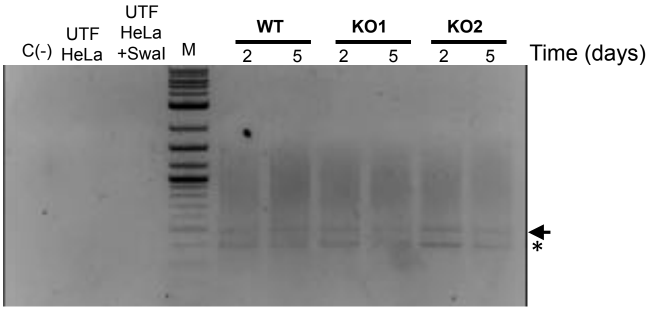

D

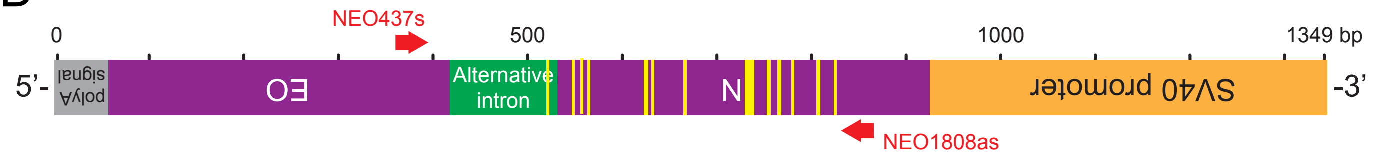

E

RNase H2	Total analyzed	Mutated	% Mutated
+	67	27	40
-	69	26	38

Appendix Figure S3. No increased mutation rate in *de novo* LINE-1 insertions in RNase H2 null cells

A. Schematic of retrotransposition assay using plasmid JM101/L1.3. Red arrows indicate primers, flanking the engineered intron present in *mneoI,* used in the PCR assay. Note the SwaI site in the engineered *mneoI* intron (dashed vertical line), which, when cut, prevents amplification of intron-containing PCR product.

B. Time line of the mutation detection assay.

C. PCR products separated by agarose gel electrophoresis. Products amplified using DNA isolated from WT, KO1 and KO2 cell lines after 2 or 5 days, as indicated; genomic DNA digested with SwaI prior to PCR (see A). C(-), negative control without template DNA. M, marker (1-Kb ladder, molecular weight standard). Arrow indicates the expected PCR product; * indicates product resulting from the use of cryptic splice sites in the Neo coding sequence.

D. Schematic of the spliced *mneoI* cassette, where the presence of tandem repeats are indicated using yellow boxes. The green box (labelled Alternative Intron) indicates the relative position of the cryptic intron. Red arrows indicate the relative position of primers used for PCR.

E. Mutation rate is not increased in RNase H2 deficient cells (-) when compared to RNase H2 proficient control cells (+).

Appendix Figure S4

A

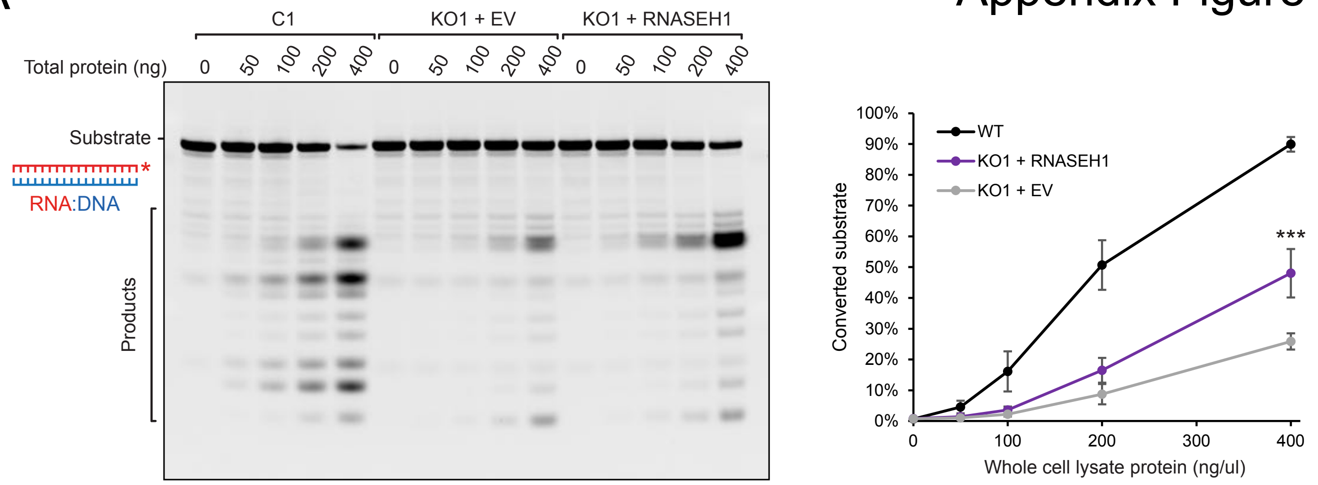

B

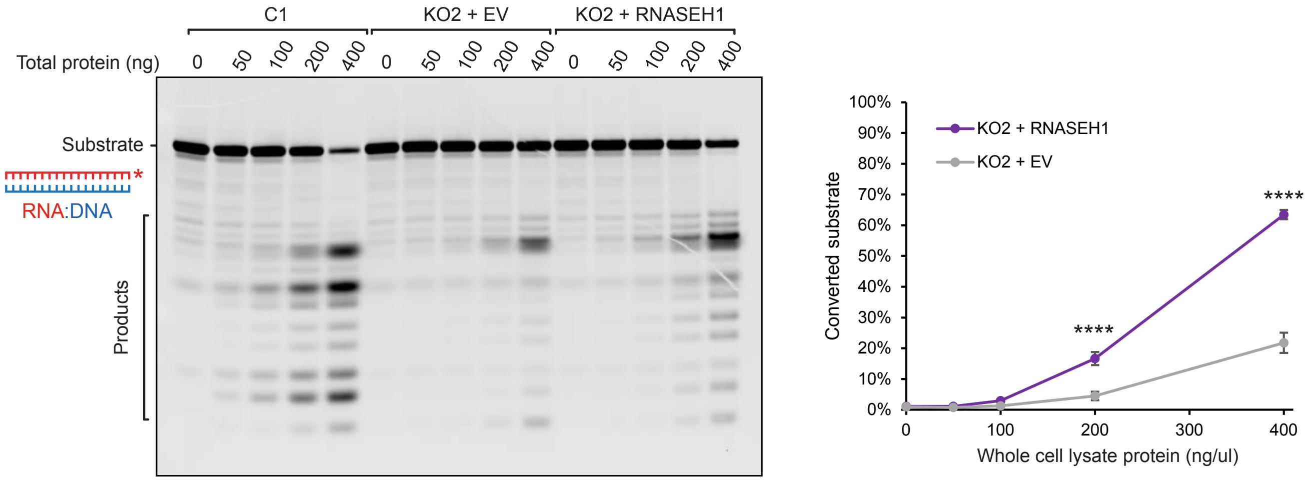

Appendix Figure S4. Increased RNase H activity against RNA:DNA heteroduplexes in RNASEH2A-KO HeLa cells complemented with human RNase H1

A, B. RNase H activity assays conducted on RNASEH2A-KO cells (**A**, KO1; **B**, KO2) complemented with human nuclear RNASEH1 (+RNASEH1) or with empty vector (+EV). RNase H activity was measured using the 18-bp RNA:DNA substrate, separating products by denaturing PAGE after incubation with lysates from the indicated cell lines using increasing amounts of total protein. Left panels, representative gels. Right panels, quantifications showing mean ± SEM of n=4 (KO1) or n=3 (KO2) independent experiments. Mean for n=3 independent experiments. Two-way ANOVA with post-hoc Bonferroni multiple comparison test shows significant increase in activity against RNA:DNA heteroduplexes in KO+RNASEH1 compared to KO+EV cells. ***, p<0.001; ****, p<0.0001
