# Supplementary material for: RNase H2, mutated in Aicardi‐Goutières syndrome, promotes LINE‐1 retrotransposition
Source: EMBO J. 2018 Jun 29;37(15):e98506. doi: 10.15252/embj.201798506 (PMC6068448; doi:10.15252/embj.201798506)
Supplement: Supplementary file 2 — Expanded View Figures PDF [file EMBJ-37-e98506-s002.pdf]

Expanded View Figures

Figure EV1. Reduced LINE-1 retrotransposition in HeLa RNase H2 null cells.

A Rationale of the retrotransposition assay using *mblastI* tagged LINE-1s (orange box with a backward BLAST label). Schematic of the retrotransposition vector JJ101/L1.3. Within the *mblastI* cassette, the orange arrow and the orange lollipop indicate the presence of a promoter and polyadenylation signal, respectively. Within L1-ORF2p, the relative position of the EN (endonuclease), RT (reverse transcriptase) and C (cysteine-rich) domains are indicated. SD and SA indicate splice donor and acceptor sites, respectively. Upon transcription from the CMV promoter located upstream of the L1, the L1 mRNA can be spliced by canonical *cis*-splicing and undergo a round of retrotransposition, resulting in the activation of the *mblastI* reporter and subsequent translation of the blasticidin deaminase protein (orange oval with blue BLAST label). In the retrotransposition event shown, the black arrows indicate the presence of target site duplications (TSDs) flanking the 5′ truncated insertion.

B Toxicity controls: Similar numbers of blasticidin-resistant colonies were generated for all cell lines after transfection with the pcDNA6.1 control vector (schematic). Representative results of transfection/selection experiments in parental HeLa cells, control clones (C1-6) and KO clones (KO1-6) are shown.

C Rationale and schematic of plasmid pYX014. With this plasmid, L1 retrotransposition activates Firefly luciferase expression. Briefly, an active human L1 is tagged with a luciferase retrotransposition indicator cassette (yellow box with a backward F-luc label). Note that the backbone of the plasmid contains an expression cassette for Renilla luciferase, to normalise for transfection efficiency (big white arrow with R-luc label). The black arrow and the black lollipop indicate the presence of a promoter and polyadenylation signal, respectively, in the F-luc cassette. Upon transfection of plasmid pXY014 in cells, the L1 mRNA is spliced by canonical *cis*-splicing and undergoes retrotransposition, resulting in the activation of the firefly luciferase reporter and subsequent translation of the F-luciferase protein (yellow star with F-luc label). On the other hand, the R-luc cassette on the plasmid can be transcribed and translated into Renilla luciferase (white star with R-luc label). In the retrotransposition event shown, the black arrows indicate the presence of TSDs flanking the 5′ truncated insertion.

D Results from retrotransposition assays conducted in HeLa parental cells, three control clones (C) and three RNASEH2A-KO clones (KO). The retrotransposition level in parental cells was set at 100%. Dots represent the mean of three technical replicates for individual clones. Lines indicate the mean of three biological replicates (C2, 4 and 5, and KO2-4) ± SEM (representative of three independent experiments). *t*-test, ***$P < 0.001$

E, F Western blot analysis of L1-ORF1p expression (in triplicate) in parental HeLa cells, a control clone (C1) and two KO clonal lines (KO1 and KO2), shows no indirect effect of RNase H2 deficiency on L1 expression. Tubulin (E) or β-actin (F) was used as a loading control.

G Quantification of Western blot from panel (C) using a LI-COR device following manufacturer's instructions.

Source data are available online for this figure.

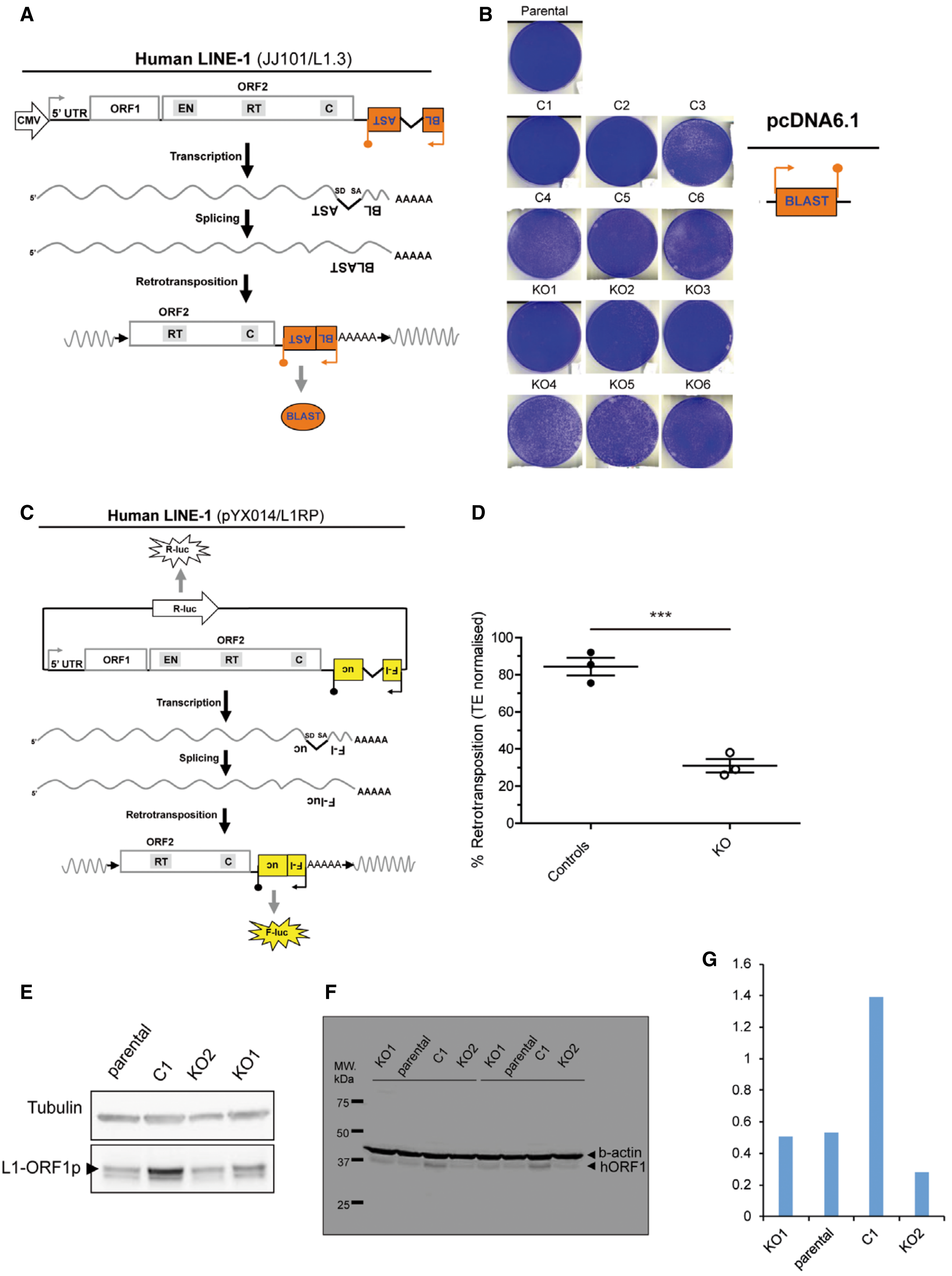

Figure EV1.

 © 2018 The Authors

Figure EV2. RNase H2 activity is required for productive LINE retrotransposition in HeLa and U2OS cells.

A Schematic of retrotransposition vectors Zfl2-2*mneoI* (zebrafish LINE-2) and JM101/L1.3 (Human L1.3). The relative position of the EN domain (endonuclease), RT domain (reverse transcriptase) and C domain (cysteine-rich), if present, is indicated. The purple box with a backward NEO label depicts the retrotransposition indicator cassette *mneoI*. Underneath, representative retrotransposition assays conducted in HeLa parental cells, control (C2-6) and RNASEH2A-KO (KO2-6) clones (used for quantifications in Figure 3C). Cells were transfected with an active human LINE-1 (WT-hL1, element L1.3), an RT-mutant human LINE-1 (RTm-hL1, D702A) or an active zebrafish LINE-2 (WT-zL2, element Zfl2-2) vector.

B Representative retrotransposition and toxicity assays conducted in parental U2OS cells, a control clone (C1) and two RNASEH2A-KO clones (KO1 and KO2). Cells were transfected with an active human LINE-1 (WT-hL1, element L1.3), an RT-mutant human LINE-1 (RTm-hL1, D702A) or an active zebrafish LINE-2 (WT-zL2, element Zfl2-2) vector. Right, quantification of WT-hL1 (dark grey bars) and WT-zL2 (light grey bars) retrotransposition in U2OS cells, with retrotransposition in parental cells for both elements set at 100% for comparison. Mean ± SD for n = 3 technical replicates (representative of three independent experiments).

© 2018 The Authors

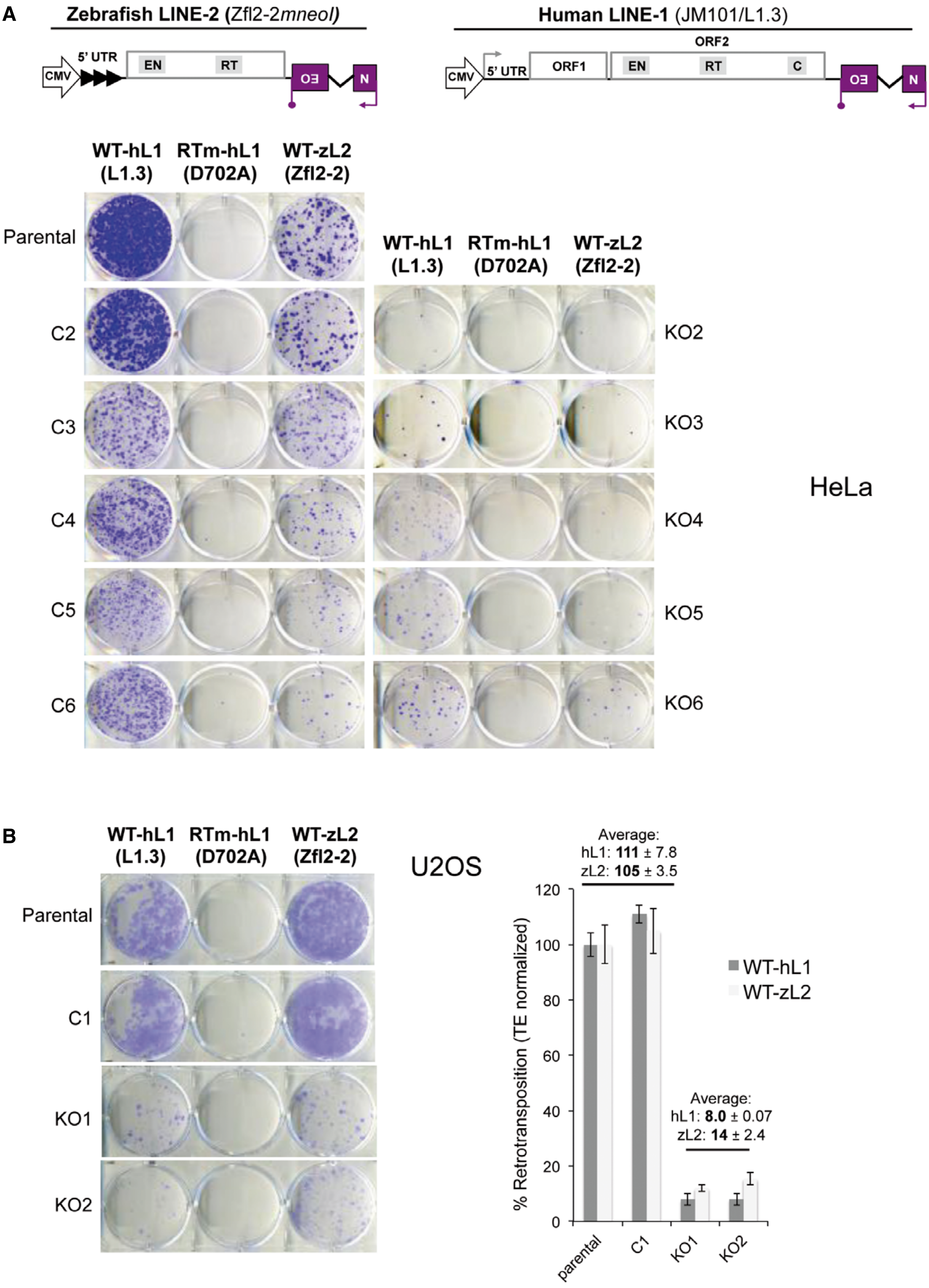

Figure EV2.

© 2018 The Authors

Figure EV3. RNase H2 overexpression facilitates L1 retrotransposition.

A Anti-V5 Western blots using lysates of HeLa (left) or U2OS (right) cells transfected with the indicated plasmids. Cells were transfected with individual plasmids expressing single RNase H2 subunits or co-transfected with plasmids expressing all three subunits using two different ratios (1:1:1 or 14:7:1). β-Actin was used as loading control. Asterisks indicate the presence of individual subunits. UTF, untransfected.

B, C Schematic of the retrotransposition vector JJ101/L1.3. Underneath, representative results from retrotransposition and toxicity assays conducted in HeLa (B) or U2OS (C) cells. Cells were transfected with vector JJ101/L1.3 or with the toxicity control vector (CTRL, pcDNA6.1) alongside an expression vector for β-arrestin (used as a negative control, −ve) or for each of the three RNase H2 subunits at the indicated ratio (RNase H2 14:7:1). Labels indicate if cells were transfected with an active human LINE-1 (WT-hL1, element L1.3), an EN-mutant LINE-1 (ENm-hL1, D205A), an RT-mutant LINE-1 (RTm-hL1, D702A), or with the toxicity control plasmid (CTRL, pcDNA6.1). Right panels, L1-WT retrotransposition quantifications, with the retrotransposition level in cells co-transfected with β-arrestin (−ve) set at 100% for comparison. Values were normalised for transfection efficiency and toxicity. Mean ± SD for n = 3 technical replicates (representative of four independent experiments). Unpaired two-sided t-test; *$P < 0.05$.

Source data are available online for this figure.

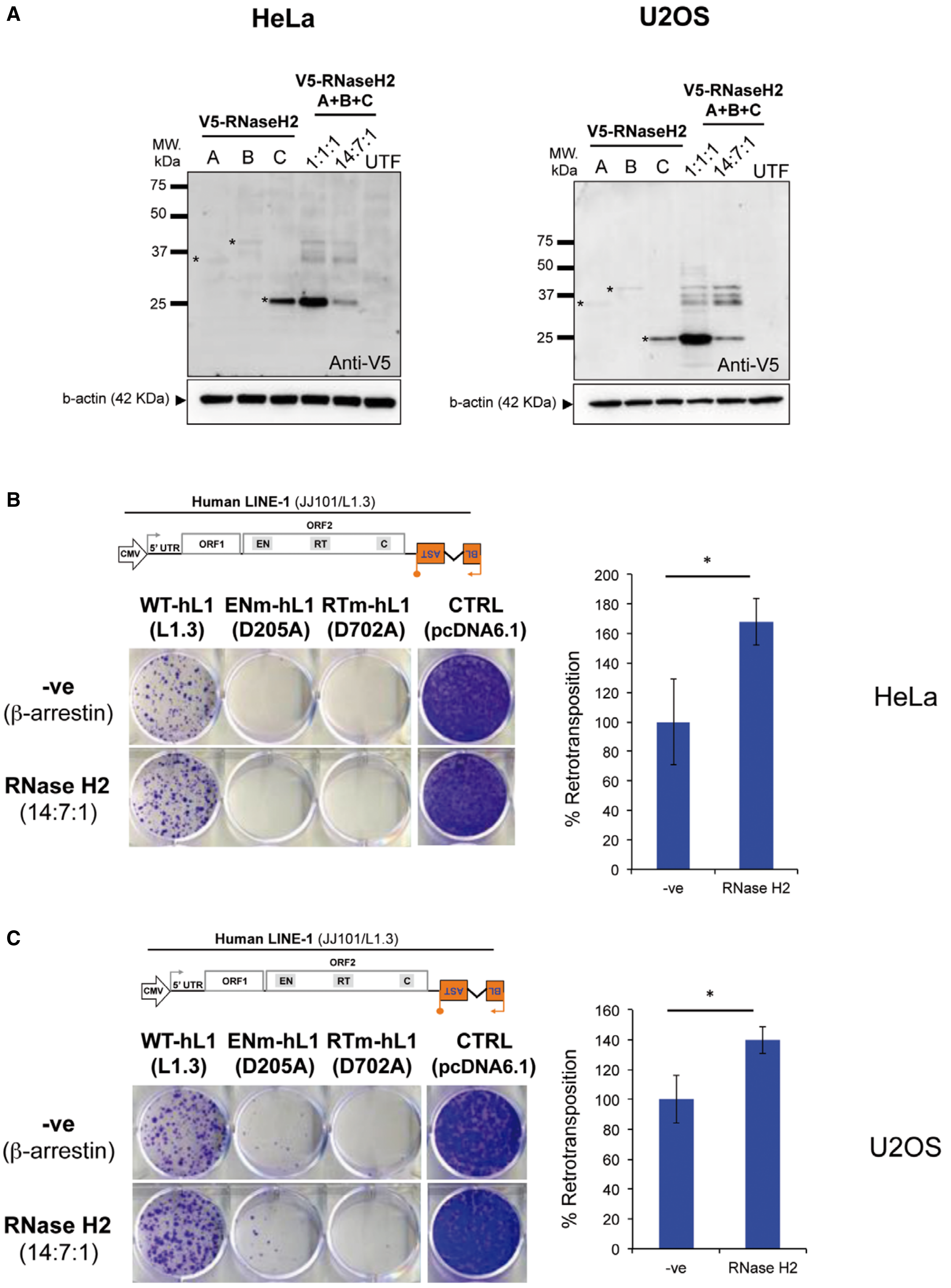

Figure EV3.

© 2018 The Authors

Figure EV4. Complementation of RNASEH2A-KO HeLa cells.

A Schematic of substrates used in RNase H activity assays. These assays either use an 18-bp RNA:DNA hybrid (left), or a short dsDNA containing a single-embedded ribonucleotide (DRD:DNA). RNASEH2A-WT can cleave both with high efficiency (++++), whereas RNASEH2A-CD (with D34A and D169A mutations) cannot cleave either (−). The separation of function mutant (SoF, with P40D and Y210A mutations) retains some activity against RNA:DNA heteroduplexes (++), but has virtually no activity against single-embedded ribonucleotides (−).

B RNase H2 SoF has reduced activity against RNA:DNA heteroduplexes and does not fully process the hybrid, even at high concentration and/or long incubation times. RNase H activity was measured using the 18-bp RNA:DNA substrate, separating products by denaturing PAGE after cleavage with RNase H2. WT, SoF and CD RNase H2 were used at 0.25 nM for 4 h (left) or 2.0 nM for 2 h (right). Note the different pattern of products generated for SoF and WT.

C As expected, the high levels of genome-embedded ribonucleotides in RNASEH2A-KO cells are rescued only by complementation with wild-type RNASEH2A (+WT), not by SoF RNASEH2A (+SoF), CD RNASEH2A-A (+CD) or the empty vector (+EV). Genomic DNA was isolated from parental cells, a control clone (C1) and the four complemented cell lines (+EV, +WT, +SoF and +CD), RNase H2 treated and separated by alkaline gel electrophoresis. Smaller fragments indicate larger numbers of embedded ribonucleotides.

D–G Representative gels (used for quantifications in Figure 5F and G) with results from RNase H activity against single-embedded ribonucleotides (D and E) and activity against RNA:DNA heteroduplexes (F and G) assays conducted with lysates from the indicated cell lines. Because RNase H1 is expressed in all of these cells, activity measured against RNA:DNA heteroduplex substrate in RNASEH2A-KO cell lysates is not completely absent. In addition, other nucleases present in the cell lysate act (non-specifically) on the substrate, causing further background activity on both substrates.

H Wild-type RNASEH2A rescues the LINE-1 retrotransposition defect in RNASEH2A-KO2 cells. Representative retrotransposition and toxicity assays conducted in RNASEH2A-KO2 cells and RNASEH2A-KO2 complemented with wild-type RNASEH2A (+WT). Cells were transfected with an active human LINE-1 (WT-hL1, element L1.3), an RT-mutant LINE-1 (RTm-hL1, D702A), or a toxicity control plasmid (CTRL, pcDNA6.1). Right panel, quantification of L1-WT retrotransposition. For comparison, the retrotransposition level in KO2 cells was set at 100%. Mean ± SD for *n* = 3 technical replicates (representative of three independent experiments).

I RNase H2 SoF has reduced RNA:DNA heteroduplex substrate affinity. Initial substrate conversion rates (V_i) by 0.1 nM recombinant RNase H2 were measured at different 18-mer RNA:DNA substrate concentrations. Mean ± SEM for *n* = 3 independent experiments. K_m and k_{cat} ± SEM were calculated in GraphPad Prism 5.04, using non-linear regression. Change for SoF compared to WT indicated between brackets.

© 2018 The Authors

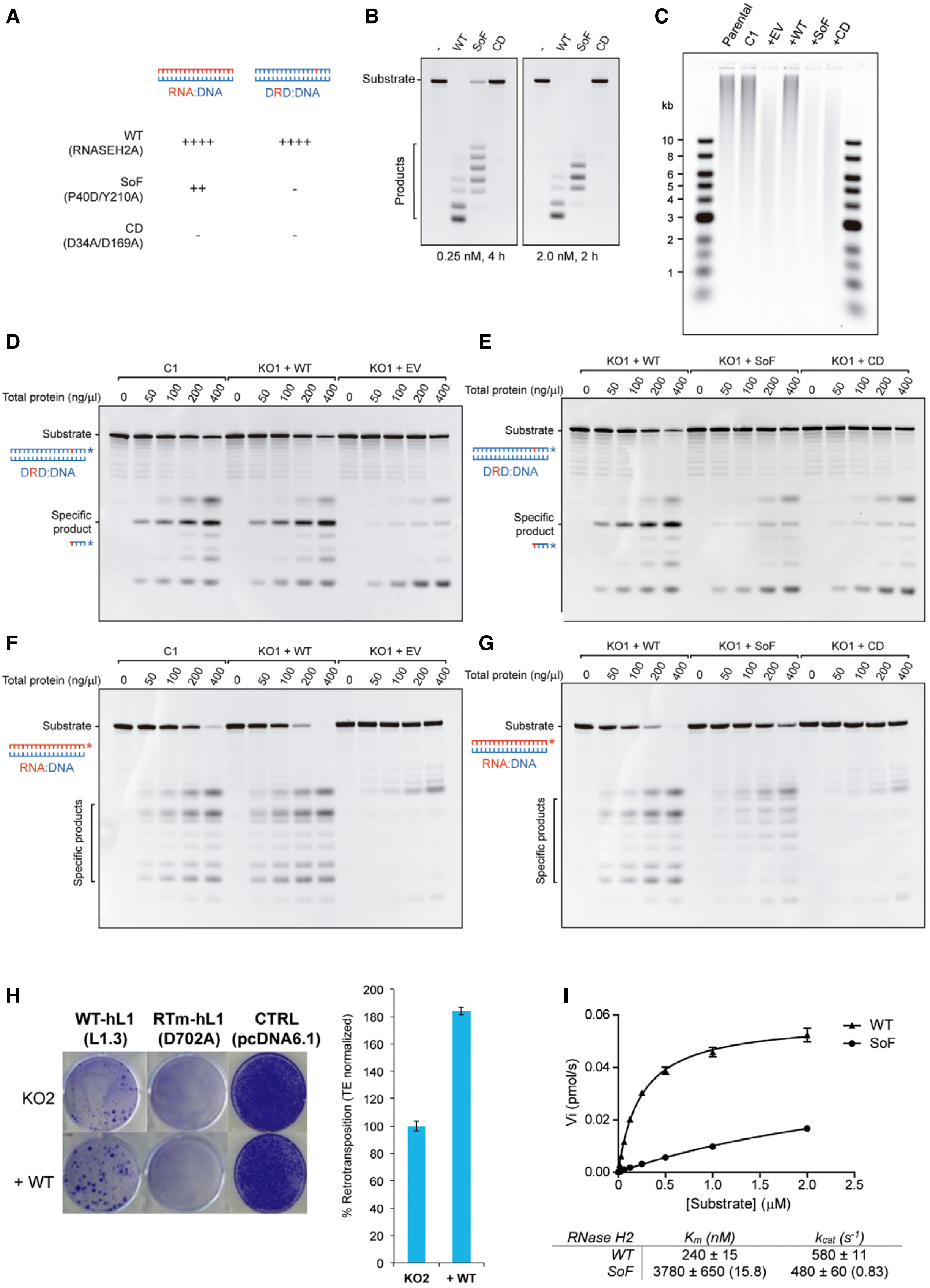

RNase H2	K_m (nM)	k_{cat} (s^{-1})
WT	240 ± 15	580 ± 11
SoF	3780 ± 650 (15.8)	480 ± 60 (0.83)

Figure EV4.

© 2018 The Authors

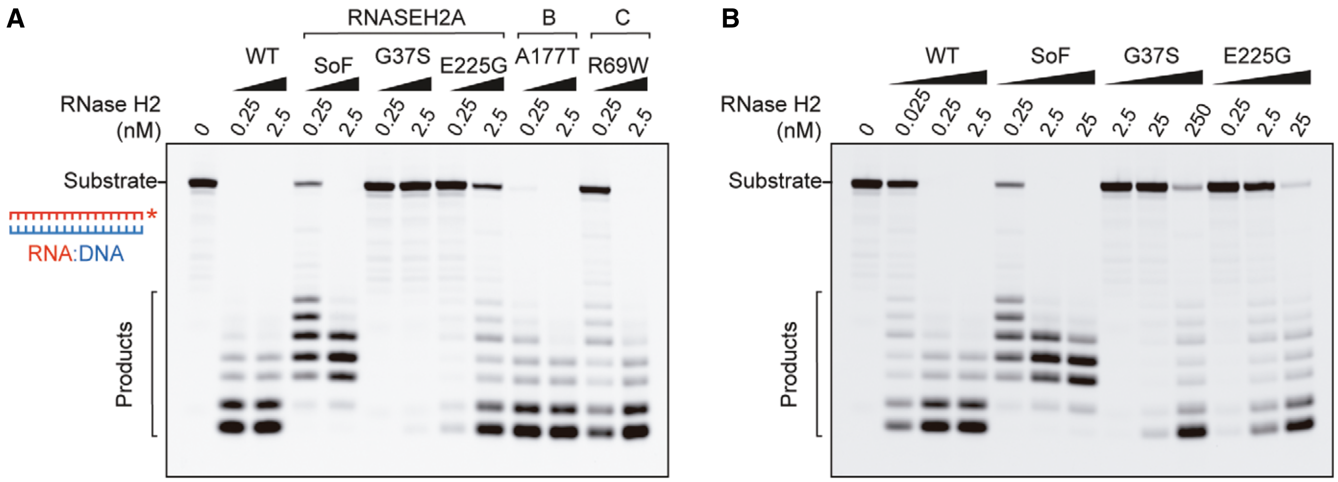

Figure EV5. Reduced enzyme activity for recombinant RNase H2 carrying AGS disease mutations.

A, B RNase H activity assays (against 18-mer RNA:DNA heteroduplex) using the indicated recombinant purified proteins. Wild type or mutant recombinant RNase H2 (RNASEH2A-P40D/Y210A, RNASEH2A-G37S, RNASEH2A-E225G, RNASEH2B-A177T and RNASEH2C-R69W) was tested using the indicated protein concentration. As previously shown (Reijns *et al*, 2011), the A177T mutation had limited impact on enzyme activity, whereas the G37S, E225G and R69W mutations all caused a substantial reduction in RNase H2 activity. Note that only RNase H2-SoF (RNASEH2A-P40D/Y210A) generates an altered cleavage pattern. Shown are representative results from three independent experiments.
