# Supplementary figures and images for: RNase H2, mutated in Aicardi‐Goutières syndrome, promotes LINE‐1 retrotransposition
Source: EMBO J. 2018 Jun 29;37(15):e98506. doi: 10.15252/embj.201798506 (PMC6068448; doi:10.15252/embj.201798506)

Source Data Figure EV1

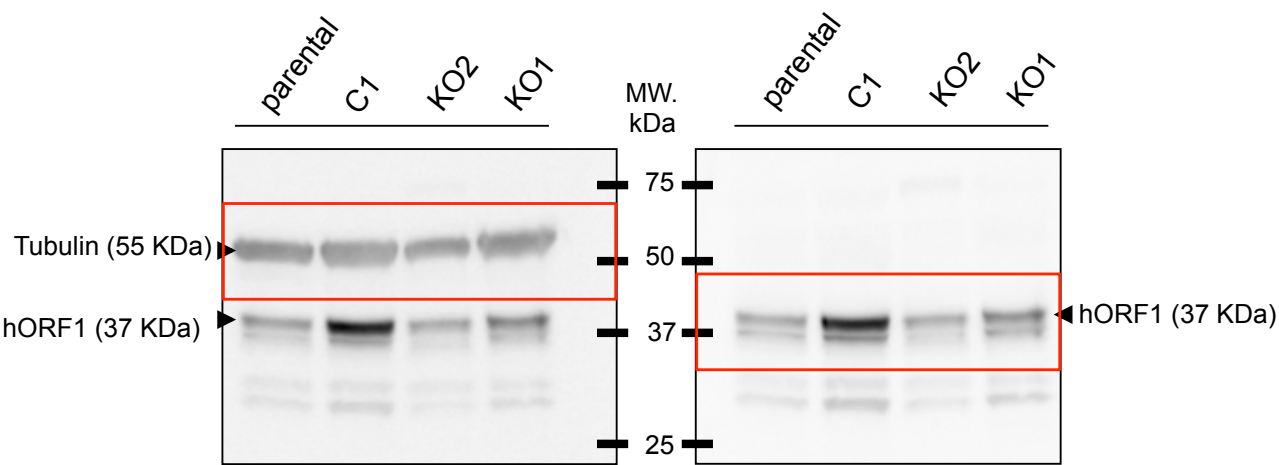

Supplement: Supplementary file 3 — Source Data for Expanded View and Appendix [file EMBJ-37-e98506-s007.zip › EMBOJ-2017-98506-SourceDataForEV1E.pdf]

Source Data Figure EV3

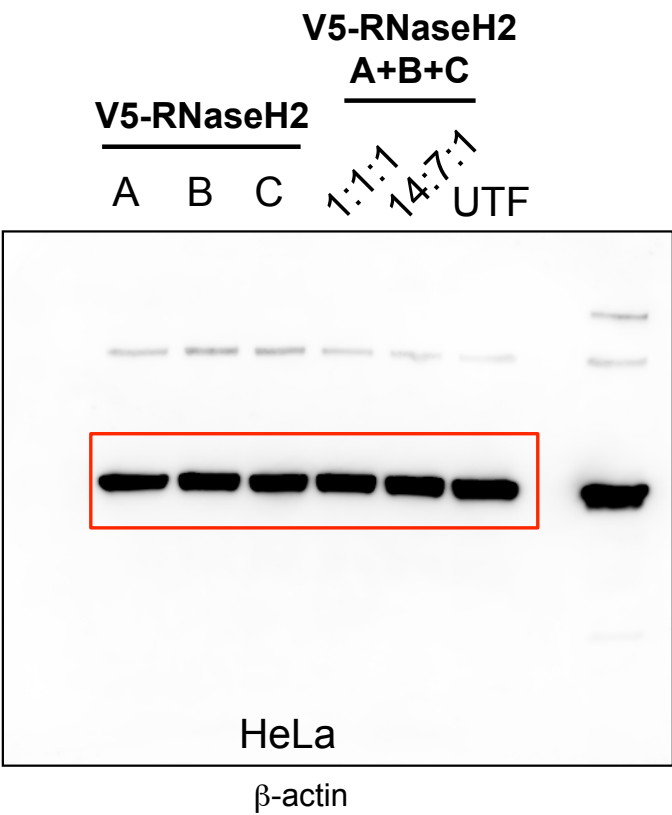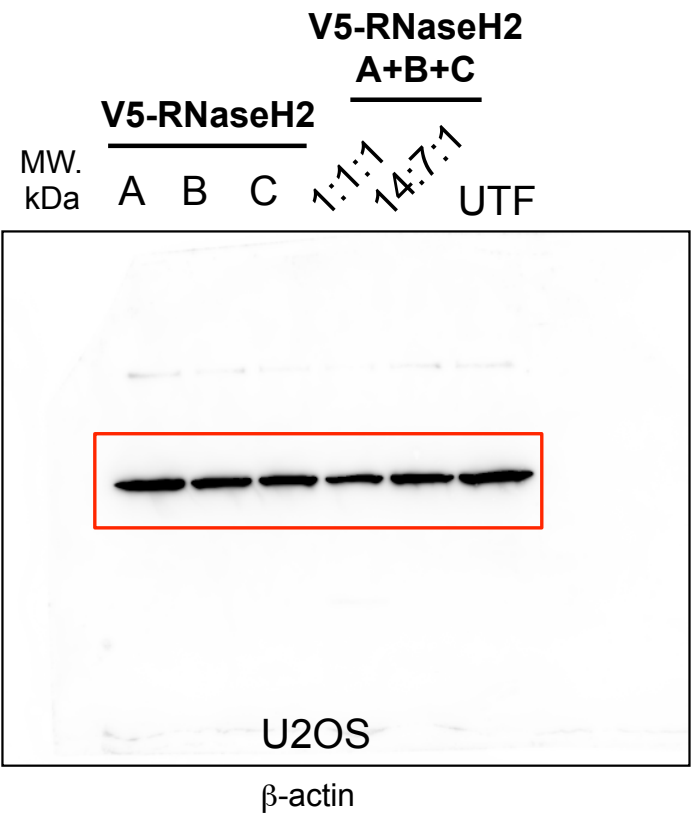

Supplement: Supplementary file 3 — Source Data for Expanded View and Appendix [file EMBJ-37-e98506-s007.zip › EMBOJ-2017-98506-SourceDataForEV3A.pdf]

Source data Appendix Fig S1

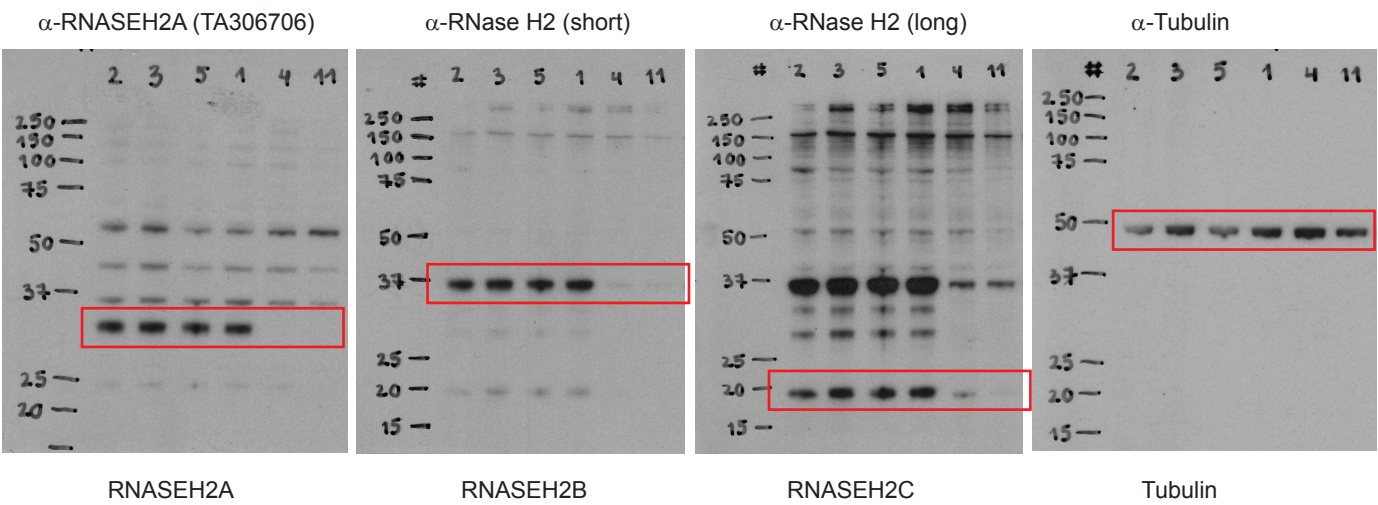

Supplement: Supplementary file 3 — Source Data for Expanded View and Appendix [file EMBJ-37-e98506-s007.zip › EMBOJ-2017-98506_SourceDataForFigureS1A.pdf]

## Source Data Fig 1

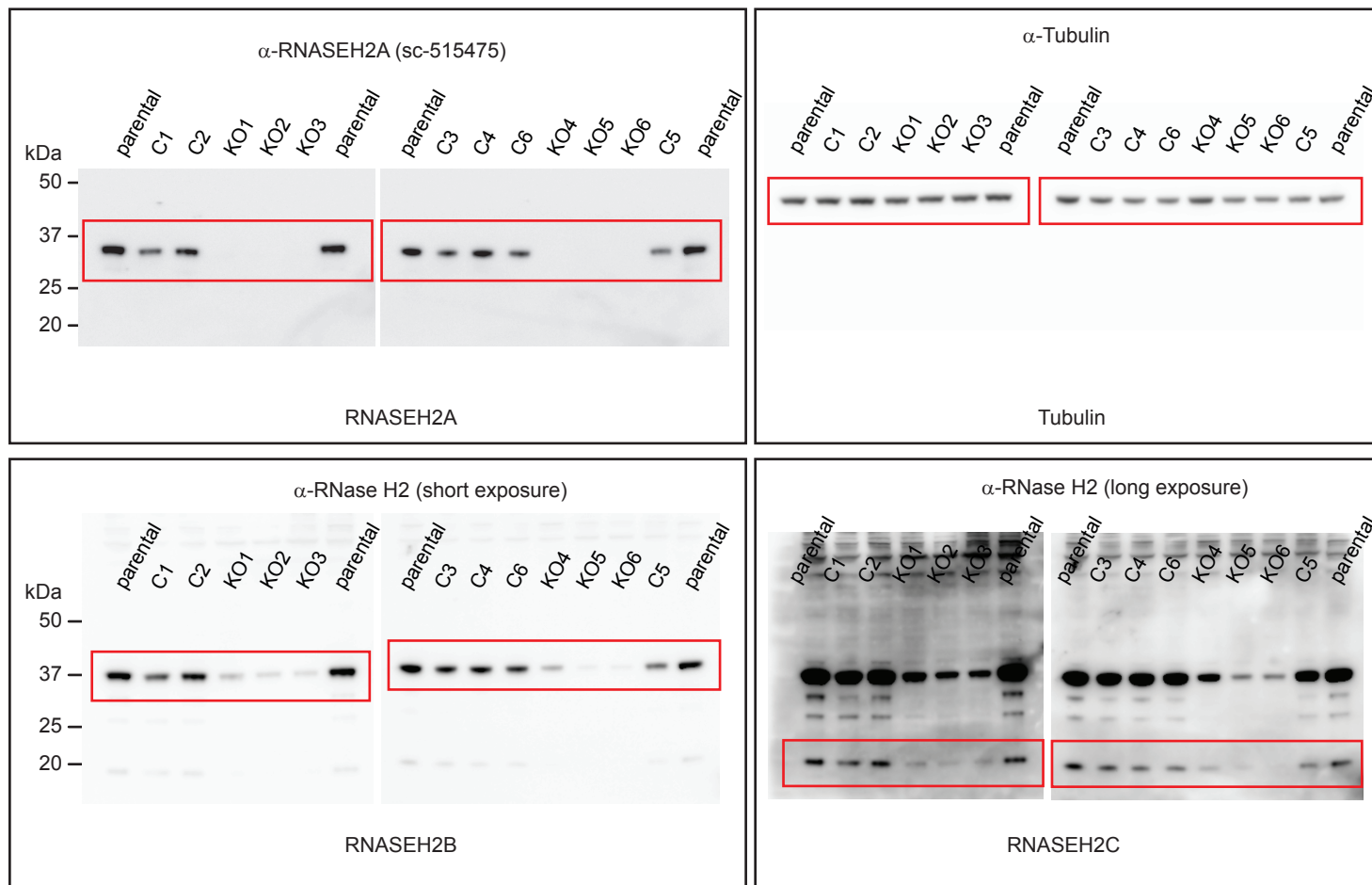

Supplement: Supplementary file 5 — Source Data for Figure 1 [file EMBJ-37-e98506-s003.pdf]

Source data Fig 2

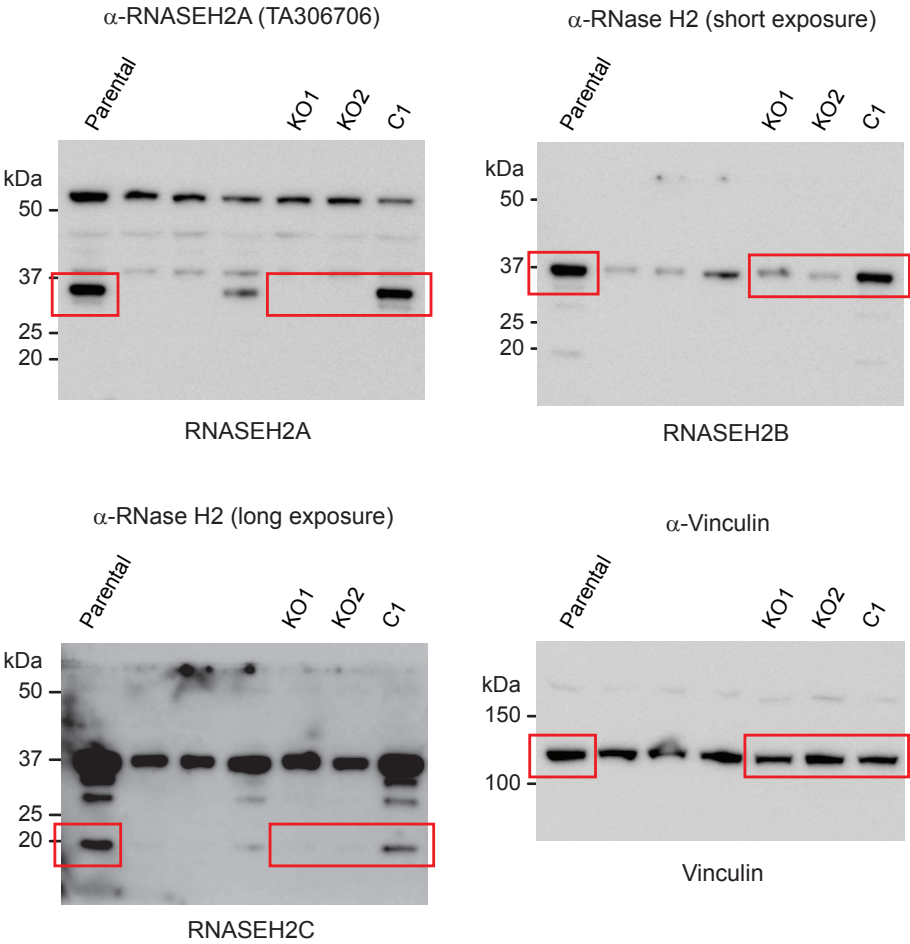

Supplement: Supplementary file 6 — Source Data for Figure 2 [file EMBJ-37-e98506-s004.pdf]

Source data Fig 5

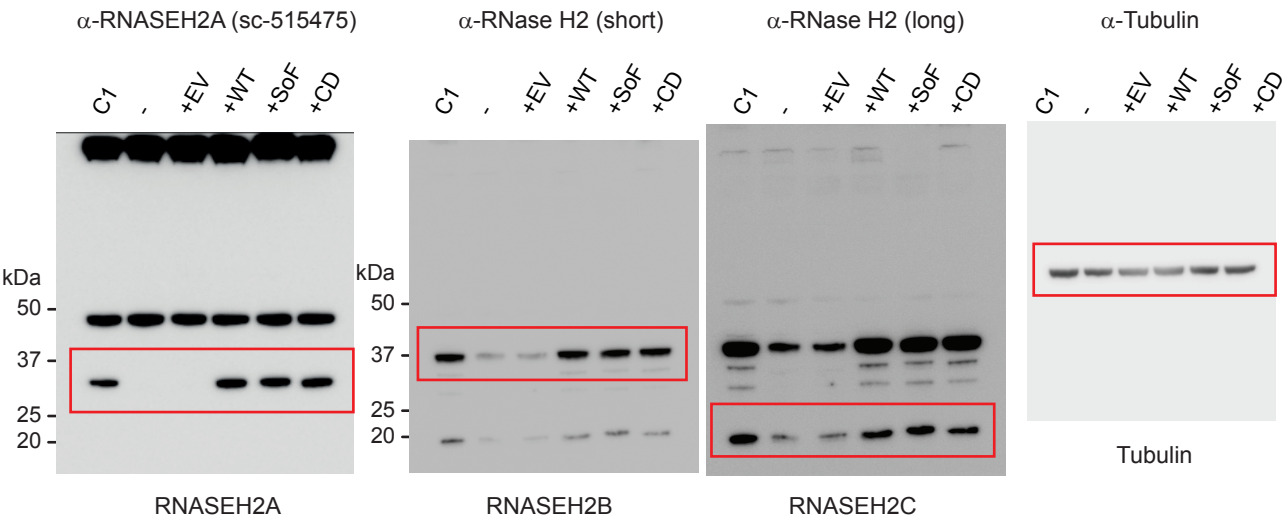

Supplement: Supplementary file 7 — Source Data for Figure 5 [file EMBJ-37-e98506-s005.pdf]

Source data Fig 7

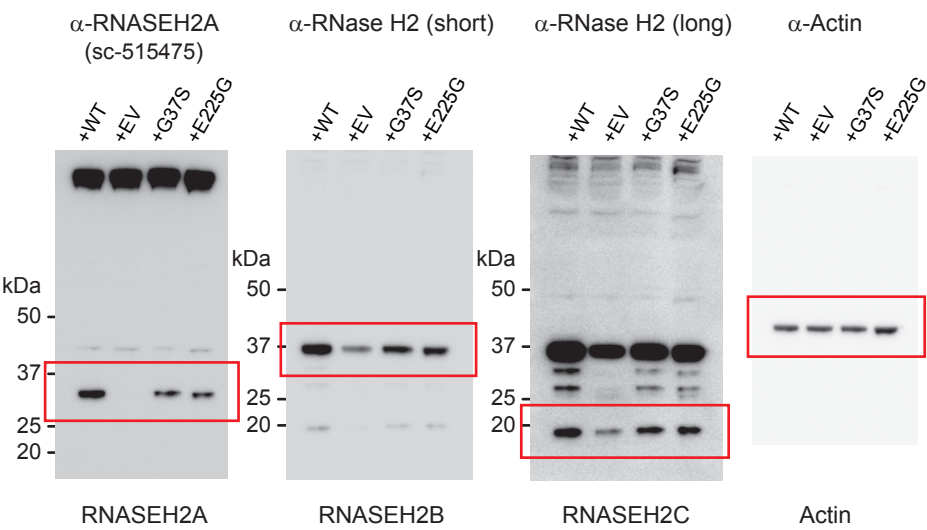

Supplement: Supplementary file 8 — Source Data for Figure 7 [file EMBJ-37-e98506-s006.pdf]
